# Pre-psychosis in later life as a risk factor for progressive cognitive decline: Findings from the IPA psychosis in neurodegenerative disease working group

**DOI:** 10.1016/j.inpsyc.2025.100094

**Published:** 2025-06-13

**Authors:** Byron Creese, Jeffrey Cummings, Corinne Fischer, Dilip Jeste, Manabu Ikeda, Kathryn Mills, Zahinoor Ismail, Clive Ballard

**Affiliations:** a Department of Psychology, College Health Medicine and Life Sciences, Brunel University of London, UK; b Chambers-Grundy Center for Transformative Neuroscience, Department of Brain Health, School of Integrated Health Sciences, University of Nevada, Las Vegas, USA; c Keenan Research Centre for Biomedical Science, St. Michael’s Hospital, Toronto, ON, Canada; d Department of Psychiatry, Temerty Faculty of Medicine, University of Toronto, Toronto, ON, Canada; e Departments of Psychiatry, Neurosciences University of California San Diego, La Jolla, USA; f Department of Psychiatry, Osaka University Graduate School of Medicine, Suita, Japan; g Department Health and Community Sciences, Faculty of Life Sciences, University of Exeter, UK; h Departments of Psychiatry, Clinical Neurosciences, Community Health Sciences, and Pathology, Hotchkiss Brain Institute and O′Brien Institute for Public Health, University of Calgary, Calgary, AB, Canada

**Keywords:** Delusions, Hallucinations, Psychosis, Dementia, Alzheimer’s

## Abstract

Pre-clinical Alzheimer’s disease (AD) has traditionally been characterized by subtle cognitive deficits alongside biomarker changes. However, emerging evidence suggests a spectrum of neuropsychiatric changes, including apathy, affective disturbances, agitation, impulse control deficits, and psychosis, may precede cognitive decline. Late-onset psychotic disorders, such as Very Late-Onset Schizophrenia-Like Psychosis (VLOSLP), differ from pre-psychosis, the latter presenting with subtle symptoms and retained insight. These subtler late-life onset symptoms are associated with incident cognitive decline, particularly in APOE4 carriers. Screening with tools such as the Mild Behavioral Impairment Checklist (MBI-C) enables the standardisation of measurement, facilitating identification of at-risk individuals. Plasma biomarkers and neuropsychological assessments further aid diagnosis and risk stratification. Understanding the link between pre-psychosis and dementia-related psychosis will be crucial, as AD with psychosis is associated with a more aggressive disease course. Identifying and treating these individuals early may improve clinical outcomes and facilitate timely intervention with disease-modifying therapies. Moreover, there remains a need to better define in what circumstances treatment interventions are indicated and what those interventions should be.

## Background

Pre-clinical Alzheimer’s disease (AD) has traditionally been characterised in terms of subtle, progressive cognitive deficits associated with biomarker changes and limited functional impairment. However, emerging evidence suggests there is a spectrum of prodromal neuropsychiatric changes that can occur before, alongside, or after the emergence of subtle cognitive deficits. These changes, broadly spanning apathy, affect, agitation, impulse control, social cognition, and pre-psychosis, have been shown in multiple independent studies to increase the risk of incident cognitive decline and dementia[1]. In this article, we discuss the concept of pre-psychosis – that is, mild late-life onset psychotic-like ideation – in the context of neurocognitive disorders. We first describe the differentiation from late-onset psychotic disorders, before discussing measurement tools, the literature linking pre-psychosis to incident cognitive decline and prodromal Alzheimer’s disease, before finally outlining possible treatment approaches.

## Differential diagnosis and assessment

Late-onset psychotic disorders are the manifestations of a variety of etiologies. Careful history and examination with laboratory investigations may be needed to rule out possible reversible causes including complications of medical illnesses and medication toxicity. Studies of the links between primary psychosis and dementia have largely focused on schizophrenia. Evidence suggests that late-onset schizophrenia (LOS) is not associated commonly with increased rates of dementia, although dementia is common in VLOSP[2]. LOS is not a prodrome of AD as progressive worsening of cognition consistent with an AD profile is uncommon. In contrast, many but not all persons with VLOSLP have some overlapping cognitive features with neurodegenerative disorders such as AD and Lewy body disease with psychosis.

One key diagnostic step is the differentiation between late-onset severe clinical presentations (e.g., Very Late Onset Schizophrenia-Like Psychosis [VLOSLP] and similar syndromes) and the more subtle presentation of emerging symptoms of pre-clinical AD. The known major differences between the two in terms of psychotic phenomenology are described in Box 1. In terms of cognitive deficits VLOSLP is associated with a range of deficits, including working memory, attention, language, risk-taking, impulse control, and visuospatial ability, which have been described in detail elsewhere [3,4]. Amongst individuals with pre-psychosis, the cognitive deficits are more subtle but - consistent with some transdiagnostic cognitive substrates - appear to be centred on recent memory, reasoning, processing speed, attention, and response inhibition (as measured by tests of grammatical reasoning and Stroop) rather than working memory, decision making, and impulse control; one study has shown that these deficits are not observed in individuals with mild affective symptoms suggesting a specificity to psychotic-like symptomology [5,6].

The distinction between these two syndromes has important implications for appropriate clinical management (whether they are part of a spectrum remains to be determined). First and most obviously, more severe clinical symptoms will carry a treatment imperative in themselves, and that treatment may differ if aetiology is known. There is also a possibility of delayed dementia diagnosis among patients with pre-psychosis due to misdiagnosing underlying neurodegenerative disease a primary psychotic disorder, which will be even more vital to avoid with the emergence of disease-modifying therapies. Plasma biomarkers will play a key role in the diagnosis and treatment of these individuals.

In contrast, people experiencing pre-psychosis may not be symptomatic enough to be in contact with health services. There has been increasing focus on the early identification of people at the pre-clinical stages of AD or other dementias, but this work has largely focussed on identifying people with subtle cognitive deficits in combination with changes in AD-related biomarkers.

Longitudinal cohort studies (discussed in more detail below) highlighting the increased risk of progressive cognitive decline and dementia amongst people with proe-psychosis suggest that there are potential benefits to screening in the community and primary care with simple screening instruments.

People with pre-psychosis represent a group of individuals that can be identified with a simple self or informant-rated questionnaire that can easily be administered through a digital platform or in a primary care setting, enabling further neuropsychological, clinical, and biomarker evaluation of these individuals. One such scale, the Mild Behavioral Impairment Checklist (MBI-C), provides a standardised framework in which to operationalise mild, late-onset but sustained abnormal thoughts and perceptions relevant to dementia risk[7]. Self- and study-partner reports of psychosis or pre-psychosis are not strongly correlated with each other, but both are associated with cognitive deficits [6,8,9]. This suggests, at least amongst individuals with normal cognition, there is retained insight into symptoms, but it is important to gather data from both self and informant reports or individuals at risk of decline may be missed, mirroring findings in younger adults [10,11].

## Epidemiological and etiological links to neurocognitive disorders

Whilst clinically diagnosed later-life onset psychotic disorders are an established risk factor for dementia [12–16], there is evidence from two recent independent analyses that the risk for dementia and incident cognitive decline extends to milder symptom profiles of pre-psychosis, such as newly occurring overvalued pre-delusional ideas. In one study, incident dementia was evaluated in 3704 cognitively normal participants (mean age 73) with or without pre-psychosis from the National Alzheimer Coordinating Centre study. Participants with neurodevelopmental, neurological, and/or longstanding psychiatric disorders were excluded. Psychosis was assessed using the Neuropsychiatric Inventory Questionnaire, with pre-psychosis operationalized as an NPI-Q score > 0 on either the delusion or hallucination items, present across two consecutive visits. Over 10 years, the dementia incidence was 4-fold higher in people reporting persistent pre-psychosis than those with no neuropsychiatric syndromes[17].

Another study used 5-year longitudinal data from 2750 PROTECT UK study participants over 50. Genotype data were available and the IQCODE was used to assess incident impairment. The MBI-C was used to identify individuals with pre-psychotic symptoms, with three questions covering overvalued pre-delusional ideas (paranoid, harm, and grandiose-type), and two questions covering hallucinations (visual and auditory). Overall, 251 (9.1 %) met the criteria for pre-psychosis (234 overvalued pre-delusional ideas, 20 hallucinations – including 3 individuals with both symptoms). The cumulative incidence of decline to IQCODE > 3.6, the threshold indicative of dementia, was 4-fold higher in people with pre-psychosis. In APOE ε4 carriers, MBI-psychosis had a 7.3-fold greater hazard than No Psychosis[8]. This increased risk of progressive cognitive decline in carriers of the *APOE4* allele with pre-psychosis raises important questions regarding the utility of genetic testing in people with this pattern of symptoms.

Etiologically, a clearer link between psychosis and AD comes from a secondary analysis of randomized controlled trial (RCT) data showing increased ptau181 levels over time in people with AD psychosis [18]. There is also data suggesting that individuals meeting the overall threshold for Mild Behavioural Impairment (based on the MBI-C assessment across a broader range of neuropsychiatric symptoms) are significantly more likely to be biomarker positive for AD, both in terms of amyloid and tau markers [19,20]. Further work is, however, needed to understand the specific biomarker profile of pre-psychosis.

## Future considerations and candidate treatments

Randomized, double-blind, placebo-controlled clinical trials of antipsychotics in LOS are sparse. In VLOSLP, amisulpride showed moderate efficacy in a RCT [21]. In LOS and VLOSLP, the choice of antipsychotic medication is usually determined by the risks of specific side effects, e.g., neurological versus metabolic side effects [2]. Given the potential detrimental impacts of atypical antipsychotics on health, mortality, and cognitive function, these agents should be avoided in people with pre-psychosis [22–24]. There are several other potential alternative treatment options. Psychological approaches, such as Cognitive Behavioural Therapy, have been effective in the treatment of functional psychosis and will be an important treatment to evaluate in clinical trials in people with pre-psychosis [25]. With regard to pharmacological treatment approaches, the 5HT2A inverse agonist pimavanserin is an effective treatment for Parkinson’s psychosis and Dementia-Related Psychosis, and does not have a detrimental impact on cognition or motor functioning [26–28]. Pimavanserin or other agents in this class are, therefore, potential treatment candidates that merit evaluation in clinical trials. In an early study, the muscarinic agonist xanomeline improved both cognition and psychosis but was poorly tolerated [29]. A new combination of xanomeline with trospium appears to have improved tolerability and exhibits antipsychotic benefits in people with schizophrenia (where it is FDA-approved), [30]. There are now several agents from this class moving forward into clinical trials in people with AD psychosis and pre-psychosis may be another high-priority indication for randomized clinical trials.

The link between pre-psychosis and psychosis in people with established dementia is important to understand. The economic and social impact of psychosis in dementia is considerable, and there are few effective treatment options [31,32]. Symptoms that are part of the dementia prodrome and are sequalae of neurodegenerative disease could be expected to progressively worsen as a direct result of the disease or secondary to deteriorating cognition, but so far, no empirical studies have tested this hypothesis. There are studies suggesting that AD associated with psychosis has a more aggressive pattern of decline, even before the onset of the psychotic symptoms [33,34]. It will therefore be important to understand if individuals exhibiting pre-psychosis that develop dementia experience a more rapid disease course. Further biomarker studies are important to better understand what proportion of these individuals have biomarker changes indicative of AD and, indeed, other dementias such as dementia with Lewy bodies, where psychosis is an even more prominent part of the clinical presentation[35].

In conclusion, pre-psychosis defines a late-life onset syndrome characterised by primarily mild delusion-like or overvalued ideation with retained insight. It can be identified with simple screening questionnaires (for example as described by Mild Behavioural Impairment framework), is associated with a marked increase in the risk of progressive cognitive decline, and identifies a group of individuals where early pre-clinical diagnosis of dementia could be substantially improved and where there may be emerging treatment opportunities.

## Box 1 Differences between late-onset schizophrenia-like syndromes and pre-psychosis.

**Table:**

Very Late and Late-onset Schizophrenia-Like Syndromes	Pre-Psychosis
Delusions (can be detailed and elaborate): Paranoia, jealousy, partition and misidentifications (in a small proportion of people)	Delusions: simple, non-systematised paranoid thoughts (persecutory; harm). May be overvalued ideas rather than fixed delusions.
Visual and auditory hallucinations	Hallucinations in any modality very rare
Limited insight	Insight often retained
